# Is acupuncture combined with repeated transcranial magnetic stimulation more effective in improving upper limb motor dysfunction after stroke? A systematic review and meta-analysis of randomized controlled trials

**DOI:** 10.3389/fneur.2025.1575879

**Published:** 2025-06-02

**Authors:** Minghui Yan, Ying Luo, Yanling Hou, Zhiying Wang, Qiguang Yang

**Affiliations:** ^1^Rehabilitation Department, Changchun Hospital of Chinese Medicine, Changchun, China; ^2^Psychological Sixth Therapeutic Area, Changchun Sixth Hospital, Changchun, China; ^3^Orthopedics, Changchun Hospital of Chinese Medicine, Changchun, China

**Keywords:** acupuncture, repeated transcranial magnetic stimulation, stroke, upper limb motor dysfunction, meta-analysis

## Abstract

**Background:**

Upper limb motor dysfunction is a common sequela of stroke, which adversely affects patients’ quality of life and ability of daily living. Although acupuncture and repeated transcranial magnetic stimulation (rTMS) can improve this symptom, it is uncertain whether the combined application of the two treatments can enhance the therapeutic effect.

**Objective:**

Through systematic review and meta-analysis, this study discusses the improvement effect of acupuncture combined with rTMS on upper limb motor dysfunction after stroke.

**Methods:**

We searched PubMed, Cochrane Library, Embase, Web of Science, China National Knowledge Infrastructure (CNKI), Chinese Scientific Journals Database (VIP), Wanfang Database, and Chinese Biomedical Literature Service (CBM) for randomized controlled trials of acupuncture combined with rTMS for the treatment of upper limb motor dysfunction after stroke, and performed a screening process according to the inclusion and exclusion criteria. The data were screened, extracted, and analyzed using RevMan (version 5.4) software for Meta-analysis.

**Results:**

A total of 21 papers involving 1,550 patients were included. The results of the Meta-analysis showed that the combination therapy was superior to acupuncture alone and rTMS alone in improving FMA-UE (acupuncture: MD = 7.55, 95%CI: 4.18 ~ 10.92, I^2^ = 97%, *p* < 0.00001; rTMS: MD = 9.74, 95%CI: 6.41 ~ 13.07, I^2^ = 98%, p < 0.00001); combination therapy was superior to acupuncture alone and rTMS alone in improving MBI (acupuncture: MD = 6.43, 95%CI: 4.07 ~ 8.78, I^2^ = 61%, *p* = 0.01; rTMS: MD = 9.49, 95%CI: 7.52 ~ 11.47, I^2^ = 39%, *p* = 0.12); combination therapy was more effective in improving MAS compared to acupuncture (MD = −0.55, 95% CI: −0.69 to −0.41, I^2^ = 0%, *p* = 0.61); combination therapy was more effective in improving NIHSS compared to rTMS (MD = −3. 14, 95%CI: −4.79 to −1.5, I^2^ = 74%, *p* = 0.02).

**Conclusion:**

Acupuncture combined with rTMS is more effective than acupuncture or rTMS intervention alone in improving upper extremity motor function and daily living ability and improving neurological damage after stroke.

## 1 Introduction

Stroke is a major health hazard and the second leading cause of death and the leading cause of disability worldwide (1). It is also the leading cause of disability. More importantly, many stroke survivors often suffer from a variety of sequelae, such as motor dysfunction, dysphagia, insomnia, and so on (2). Upper limb motor dysfunction is one of the most common sequelae, with a prevalence of 55–75% in stroke patients (3). A study showed that only 5–20% of patients had a good recovery of upper limb function 6 months after stroke (4). This adversely affects the patient’s condition and quality of life. For stroke patients, intensive rehabilitation is the main tool to improve upper limb dysfunction. Although different physical therapies have been developed, such as constraint-induced movement therapy and mirror therapy, the recovery of upper limb function is still very slow despite the application of various rehabilitation therapies (5, 6). The development and formulation of new therapeutic options and strategies is warranted.

Acupuncture is a traditional therapeutic method with a long history, which has been widely used in many diseases and is easy to operate and shows rapid effects. In recent years, the application of acupuncture in post-stroke sequelae has gradually become popular and achieved good clinical results (7–10). It is recognized by the World Health Organization as a complementary and alternative therapy for stroke (11, 12). Acupuncture is often combined with repeated transcranial magnetic stimulation (rTMS) in the treatment of upper limb motor dysfunction after stroke (13, 14). Currently, there have been systematic reviews providing evidence that acupuncture and rTMS can improve upper limb motor dysfunction after stroke, respectively (7, 9, 15). But there is still a lack of evidence-based evidence of acupuncture combined with rTMS on upper limb motor dysfunction after stroke. Therefore, the aim of this study was to evaluate the efficacy of acupuncture combined with rTMS in the treatment of post-stroke upper extremity motor dysfunction and to provide a reference for the clinical treatment of this disease.

## 2 Materials and methods

The analysis was performed in accordance with the PRISMA statement and registration with PROSPERO was completed prior to conducting this study (registration number: CRD42024548360).

### 2.1 Search scope and search strategy

This study searched 8 databases including PubMed, Cochrane Library, Embase, Web of Science, China National Knowledge Infrastructure (CNKI), China Science Journal Database (VIP), Wanfang Database and China Biomedical Literature Service System (CBM), the language of publications was limited to English and Chinese, and the search was conducted until May 19, 2024. The search was conducted using a Mesh words union free words search, and the main search terms included “Stroke,” “Upper Extremity” “Transcranial Magnetic Stimulation” “Acupuncture.” Specific search strategies are demonstrated in Table 1 (using Pubmed as an example).

**Table 1:** Search strategy (Pubmed as an example).

Rank	Search formula	Result
#1	"Cerebrovascular Disorders"[Mesh] OR "Basal Ganglia Cerebrovascular Disease"[Mesh] OR "Brain Ischemia"[Mesh] OR "Carotid Artery Diseases"[Mesh] OR "Carotid Artery Diseases"[Mesh] OR "Intracranial Arterial Diseases"[Mesh] OR Cerebral Arterial Diseases OR "Intracranial Embolism and Thrombosis"[Mesh] OR "Intracranial Hemorrhages"[Mesh] OR "Basal Ganglia Hemorrhage"[Mesh] OR "Stroke"[Mesh]	460570
#2	stroke OR poststroke OR post-stroke OR cerebrovasc* OR brain vasc* OR Cerebral Infarction OR cerebral vasc* OR cva OR cvas OR apoplex* OR SAH OR ICH OR ICHs	736766
#3	(brain OR cerebr* OR cerebell* OR vertebrobasil* OR hemispher* OR intracran* OR intracerebral OR infratentORial OR supratentORial OR middle cerebr* OR mca OR anteriOR circulation) AND (isch?emi* OR infarct* OR thrombo* OR emboli* OR occlus* OR hypoxi*)	266674
#4	(brain* OR cerebr* OR cerebell* OR intracerebral OR intracran* OR parenchymal OR intraparenchymal OR intraventricular OR infratentORial OR supratentORial OR basal gangli* OR putaminal OR putamen OR posteriOR fossa OR hemispher*) AND (h?emORrhag* OR h?ematoma* OR bleed*)	27768
#5	#1 OR #2 OR #3 OR #4	954900
#6	("Upper Extremity"[Mesh]) OR "Arm"[Mesh]	195594
#7	Extremities, Upper OR Upper Extremities OR Membrum superius OR Upper Limb* OR Limb, Upper OR Limbs, Upper OR Extremity, Upper	256627
#8	#6 OR #7	256627
#9	("Transcranial Magnetic Stimulation"[Mesh]) OR "Transcranial Direct Current Stimulation"[Mesh]	21967
#10	Magnetic Stimulation?, Transcranial OR Stimulation?, Transcranial Magnetic OR Transcranial Magnetic Stimulations OR Transcranial Magnetic Stimulation, Single Pulse OR Transcranial Magnetic Stimulation, Paired Pulse OR Transcranial Magnetic Stimulation, Repetitive	26124
#11	#9 OR #10	30497
#12	Acupuncture[Mesh] OR Acupuncture Therapy[Mesh] OR Acupuncture Points[Mesh]	32491
#13	Acupuncture Treatment* OR Treatment, Acupuncture OR Therapy, Acupuncture OR Acupotomy OR Acupotomies OR Electroacupuncture OR Manual acupuncture OR Scalp Acupuncture	44087
#14	#12 OR #13	44304
#12	#5 AND #8 AND #11 AND #14	15

### 2.2 Inclusion and exclusion criteria

The inclusion criteria for this study were set according to the PICOS (Population, Intervention, Comparison, Outcome, and Study Design) framework. The specific inclusion criteria were (1) The subjects were patients with a clear diagnosis of stroke and concomitant motor dysfunction of the upper limbs; (2) The experimental group was treated with acupuncture combined with rTMS, which included hand acupuncture, electroacupuncture, as well as special acupuncture methods such as abdominal and head acupuncture; (3) The control group was intervened with either acupuncture alone or rTMS alone; (4) The mainoutcome indexes included the Upper Extremity Fugl-Meyer (FMA-UE) scale, Modified Barthel Index (MBI), and secondary outcome indicators included Motor Assessment Scale (MAS), National Institute of Health Stroke Scale (NIHSS); (5) The study type was randomized controlled study.

Exclusion criteria: (1) Studies in which subjects had an unclear diagnosis or other concomitant diseases that affected the judgment of the results; (2) Studies that received other interventions at the same time; (3) Studies in which the outcome indexes were not representative; (4) Not randomized controlled studies; (5) Studies in which full text was unavailable or the data were incomplete.

### 2.3 Literatures screening and sata extraction

2 researchers independently performed literature screening and data extraction based on the inclusion and exclusion criteria, with a third researcher assessing the process if disagreements arose. The specific screening process was: (1) To exclude duplicate literatures using literature management software Endnote X9. (2) Read the titles and abstracts to exclude other types of literatures such as reviews, dissertations, conference papers, scientific and technological achievements, and literatures with irrelevant research content. (3) Read the full text to determine whether it meets the inclusion criteria.

The following information was extracted after the literatures screening was completed: (1) Basic information of the literatures: name of the study, year of publication, first author; (2) Basic information of the subjects: sample size, age, gender, duration of the disease; (3) Method of intervention, frequency of the intervention, and duration of the course of the treatment; (4) Outcome indexs (According to Cochrane recommendations, all outcome indicators that were continuous type variables were extracted based on the difference between baseline and treatment endpoints).

### 2.4 Quality assessment

We evaluated the quality of the included literatures using the Cochrane risk-of-bias tool for randomized trials (RoB 2) Assessment Tool. RoB 2 is structured into a fixed set of domains of bias, focussing on different aspects of trial design, conduct, and reporting. Within each domain, a series of questions (‘signalling questions’) aim to elicit information about features of the trial that are relevant to risk of bias. A proposed judgement about the risk of bias arising from each domain is generated by an algorithm, based on answers to the signalling questions.

### 2.5 Statistical analysis

Meta-analysis was performed using RevMan 5.4 software. Since the outcomes indexs in this study were continuous variables, mean difference (MD) and standardized mean difference (SMD) were used to represent the combined statistics and their 95% confidence intervals were calculated. I^2^ was the measure of heterogeneity, and a strong heterogeneity was indicated by I^2^ > 50%, using a random effects model. If there were multiple control groups in a study, it was categorized into multiple independent studies. We conducted subgroups analyses based on the intervention modality in the control group and analyzed bias using funnel plots.

### 2.6 Evidence certainty assessment

To assess the evidence certainty, we used the GRADE PRO online tool^1^ to perform the evaluation and followed the recommended procedures for grading (high, moderate, low, very low).

## 3 Results

### 3.1 Search results

**Figure 1:**
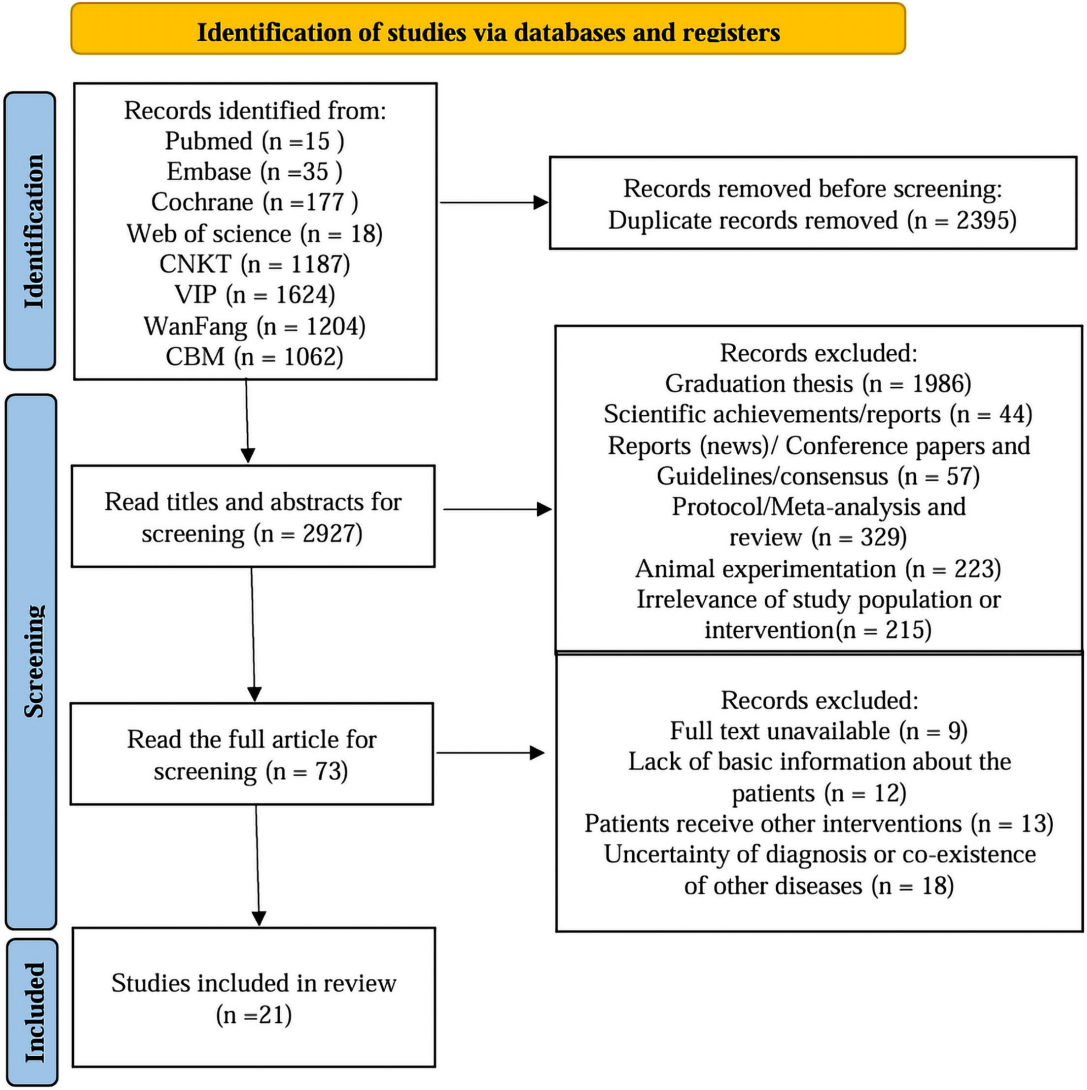
Literature screening process.

A total of 5,322 documents were retrieved from the database and 2,927 documents remained after excluding duplicates. After further screening by reading the titles and abstracts, 73 pieces of literature remained. By reading the full text of the 73 documents, 21 documents were finally screened to meet the inclusion conditions, including 2 English documents and 19 Chinese documents. The specific screening process of the literature is shown in Figure 1.

### 3.2 Basic characteristics of the included literatures

Of the 21 papers included, 8 had multiple control groups set up, so they were set up as 2 studies, and a total of 29 studies were applied to the meta-analysis (2–4, 7–24). The basic characteristics of the included literatures are shown in Table 2.

**Table 2:** The basic characteristics of the included literatures.

No.	Author’s name	Sample size	Sex (male/female)	Age (Mean±SD)	Duration of disease (days/weeks/months)	Intervention methods	duration of treatment (days/weeks/months)	Outcome indexs
1	Yang (14)	I:51 C:51	I:30/21 C:32/19	I:64.62 ± 6.54 C:65.36 ± 6.44	I:40.21 ± 5.44(d) C:41.42 ± 5.6(d)	I: Scalp acupuncture + LF-rTMS C: Scalp acupuncture	1 times/d 8w	FMA-UE, MBI, MAS
2	Zhu et al. (19)	I:34 C1:35 C2:37	I:20/14 C1:23/12 C2:25/12	I:58.74 ± 7.45 C1:57.45 ± 7.36 C2:57.12 ± 7.24	I:58.62 ± 8.14(d) C1:58.15 ± 7.65(d) C2:55.74 ± 8.32(d)	I: Scalp acupuncture + Acupuncture + LF-rTMS C1: Scalp acupuncture + Acupuncture C2: LF-rTMS	6 times/w 6w	FMA-UE, MBI, MAS
3	Wang et al. (11)	I:35 C:35	I:21/14 C:24/11	I:60.88 ± 8.28 C:64.40 ± 10.20	I:48.50 ± 14.33(d) C:45.63 ± 12.26(d)	I: Acupuncture + LF-rTMS C: Acupuncture	6 times/w 3w	FMA-UE, MBI
4	Xie et al. (16)	I:17 C:19	I:10/7 C:13/6	I:54.47 ± 9.152 C:58.42 ± 12.76	I: 20 ± 13.33(d) C:16 ± 8.89(d)	I: Acupuncture + LF-rTMS C: LF-rTMS	5 times/w 2w	FMA-UE, MBI, Ashworth, MEPL, MEPA
5	Wang et al. (12)	I:30 C:30	I:20/10 C:18/12	I:57.18 ± 12.06 C:56.43 ± 11.27	I:67.5 ± 8.76(d) C:63.6 ± 9.61(d)	I: Electroacupuncture +LF-rTMS C: LF-rTMS	5 times/w 4w	FMA-UE, MBI, MEPL, MEPA
6	Cai et al. (2)	I:40 C1:40 C2:40	I:29/11 C1:26/14 C2:25/15	I:60.9 ± 3.1 C1: 61.1 ± 3.3 C2: 60.3 ± 3.3	I:70.4 ± 8.0(d) C1: 71.2 ± 8.1(d) C2:70.1 ± 8.2(d)	I: Electroacupuncture + LF-rTMS C1: Electroacupuncture C2: LF-rTMS	5 times/w 2w	FMA-UE, MBI, MI, MEPL, MEPA, CMCT
7	Sun et al. (10)	I:40 C1:40 C2:40	I:28/12 C1:25/15 C2:27/13	I:65.83 ± 5.10 C1:65.92 ± 5.12 C2:65.80 ± 4.98	I:27.86 ± 4.32(d) C1:27.93 ± 4.29(d) C2:27.90 ± 4.30(d)	I: Scalp acupuncture + LF-rTMS C1: Scalp acupuncture C2: LF-rTMS	1 times/d 3 m	FMA-UE, NIHSS, PM, DLS,
8	Zhang et al. (17)	I:32 C:32	I:22/10 C:25/7	I:59.75 ± 8.12 C:60.03 ± 7.66	I:11.84 ± 6.03(w) C:12.19 ± 6.36(w)	I: Electroacupuncture + LF-rTMS C: Electroacupuncture	5 times/w 4w	FMA-UE, MBI, mRS, MEPA
9	Huang et al. (4)	I:22 C1:22 C2:22	I:15/7 C1:16/6 C2:14/8	I:55.73 ± 11.83 C1:55.25 ± 11.73 C2:56.14 ± 10.24	I:64.10 ± 16.89(d) C1:63.77 ± 18.42(d) C2:62.81 ± 16.74(d)	I: Acupuncture + LF-rTMS C1: Acupuncture C2: LF-rTMS	5 times/w 4w	FMA-UE, MBI, RMS, iEMG
10	Cao et al. (3)	I:30 C:30	I:13/17 C:16/14	I:56.9 ± 4.7 C:55.1 ± 3.2	I:65.2 ± 34.8(d) C:68.8 ± 26.5(d)	I: Scalp acupuncture + LF-rTMS C: LF-rTMS	6 times/w 4w	MBI, Broetz, HHFGS,
11	Ji et al. (7)	I:30 C:30	I:17/13 C:16/14	I:55.5 ± 3.7 C:55.3 ± 3.2	I:2.9 ± 0.2(m) C:2.7 ± 0.3(m)	I: Scalp acupuncture + LF-rTMS C: LF-rTMS	5 times/w 3w	FMA-UE, MBI, NIHSS
12	Wu (13)	I:28 C1:28 C2:28	I:17/11 C1:16/12 C2:15/13	I:55.74 ± 3.31 C1:55.67 ± 3.29 C2:55.59 ± 3.18	I:33.56 ± 4.11(d) C1:33.42 ± 3.12(d) C2:33.57 ± 4.62(d)	I: Acupuncture + LF-rTMS C1: Acupuncture C2: LF-rTMS	5 times/w 2w	FMA-UE
13	Li et al. (8)	I:75 C:75	I:40/35 C:42/33	I:50.26 ± 1.31 C:50.17 ± 1.25	I:3.10 ± 0.12(m) C:3.08 ± 0.15(m)	I: Acupuncture + LF-rTMS C: LF-rTMS	1 times/d 24d	FMA-UE, NIHSS, CCDI
14	Yu (15)	I:36 C:36	I:22/14 C:23/13	I:58.9 ± 6.7 C:57.6 ± 6.4	I:48.3 ± 13.5(d) C:47.5 ± 12.7(d)	I: Scalp acupuncture + Acupuncture + LF-rTMS C: LF-rTMS	I:6 times/w 4w C:5 times/w 4w	FMA-UE, MBI, Wolf
15	Zhu et al. (18)	I:20 C1:20 C2:20	I:11/9 C1:10/10 C2:9/11	I:64.23 ± 10.54 C1:65.45 ± 13.12 C2:61.00 ± 11.68	I:34.45 ± 11.61(d) C1:35.68 ± 12.73(d) C2:37.20 ± 14.74(d)	I: Acupuncture + LF-rTMS C1: Acupuncture C2: LF-rTMS	1 times/d 20d	FMA-UE, Wolf,
16	Luo et al. (9)	I:20 C:20	I:14/6 C:12/8	I:52.9 ± 14.4 C:54.1 ± 14.9	I:3.9 ± 2.6(w) C:3.7 ± 1.9(w)	I: Acupuncture + LF-rTMS C: LF-rTMS	5 times/w 4w	FMA-UE, MBI, NIHSS
17	Wang et al. (21)	I:20 C1:20 C2:20	I:11/9 C1:10/10 C2:9/11	I:62.13 ± 12.54 C1:65.45 ± 11.12 C2:60.00 ± 12.68	I: 35.45 ± 14.61(d) C1: 37.68 ± 15.73(d) C2: 33.2 ± 13.74(d)	I: Acupuncture + LF-rTMS C1: Acupuncture C2: LF-rTMS	5 times/w 4w	FMA-UE, VAS
18	Kim et al. (20)	I:11 C1:11 C2:8	I:4/7 C1:7/4 C2:5/3	I: 67.55 ± 12.53 C1:64.45 ± 14.75 C2:67.00 ± 12.92	/	I: Scalp acupuncture + LF-rTMS C1: Scalp acupuncture C2: LF-rTMS	5 times/w 3w	FMA-UE, MBI, NIHSS, FIM, EQ-5D
19	Jiang et al. (24)	I:25 C:25	I:13/12 C:17/8	I:56.72 ± 10.5 C:54.56 ± 12.68	I:2.62 ± 1.18(m) C:2.66 ± 1.12(m)	I: Electroacupuncture + LF-rTMS C: Electroacupuncture	5 times/w 4w	MAS, FMA-UE, MBI, sEMG
20	Lei et al. (22)	I:20 C:20	I:18/2 C:13/7	I:58.90 ± 9.49 C:57.70 ± 12.37	I: 1.82 ± 1.25(m) C: 1.60 ± 1.36(m)	I: Scalp acupuncture + Acupuncture + LF-rTMS C: Scalp acupuncture + Acupuncture	5 times/w 4w	MBI, FMA-UE, MAS, O_2_Hb
21	Liu et al. (23)	I:50 C:50	I:28/22 C:30/20	I:73.05 ± 6.31 C:72.37 ± 5.63	I:21.41 ± 5.61(d) C:20.21 ± 5.44(d)	I: Electroacupuncture + LF-rTMS C: Electroacupuncture	6 times/w 4w	BDNF, NGF, MAS, FMA-UE

C, Control group; CMCT, Central Motor Conduction Time; d, days; DLS, Daily living skills; EQ-5D, European Quality of Life-5 Dimensions; FMA-UE, Fugl Meyer Assessment-Upper Extremity; HHFGS, Hemiplegic hand function grading scale; LF-Rtms, Low Frequency-repetitive Transcranial Magnetic Stimulation; MAS, Modified Ashworth scale; MBI, Modified Barthel Index; sEMG, surface electromyography; O2Hb, oxyhemoglobin, MEPL, Motor Evoked Potential Cortical Latency; MEPA, Motor Evoked Potential Amplitude; m, months; NIHSS, National Institute of Health Stroke Scale; PM, Physical mobility; VAS, Visual Analog Scale; Wolf Motor Function Test; BDNF, brain-derived neurotrophic factor; NGF, nerve growth factor; I, Intervention group; w, weeks.

A total of 1,550 patients were enrolled in 29 studies, of whom 933 (60.19%) were male and 617 (39.81%) were female. In terms of diagnosis, all researches did not differentiate between types of stroke, and in terms of intervention, all researches used LF-rTMS. In terms of acupuncture intervention, 8 papers used scalp acupuncture, 12 papers used body acupuncture, 5 papers used electroacupuncture, and in terms of characteristic acupuncture techniques, 3 papers used “Kai qiao xing shen” acupuncture, 1 paper used “Jin’s three-needle therapy “, and 1 paper used “contracted three-needle therapy.”

### 3.3 Analysis of the quality of the included literatures

**Figure 2:**
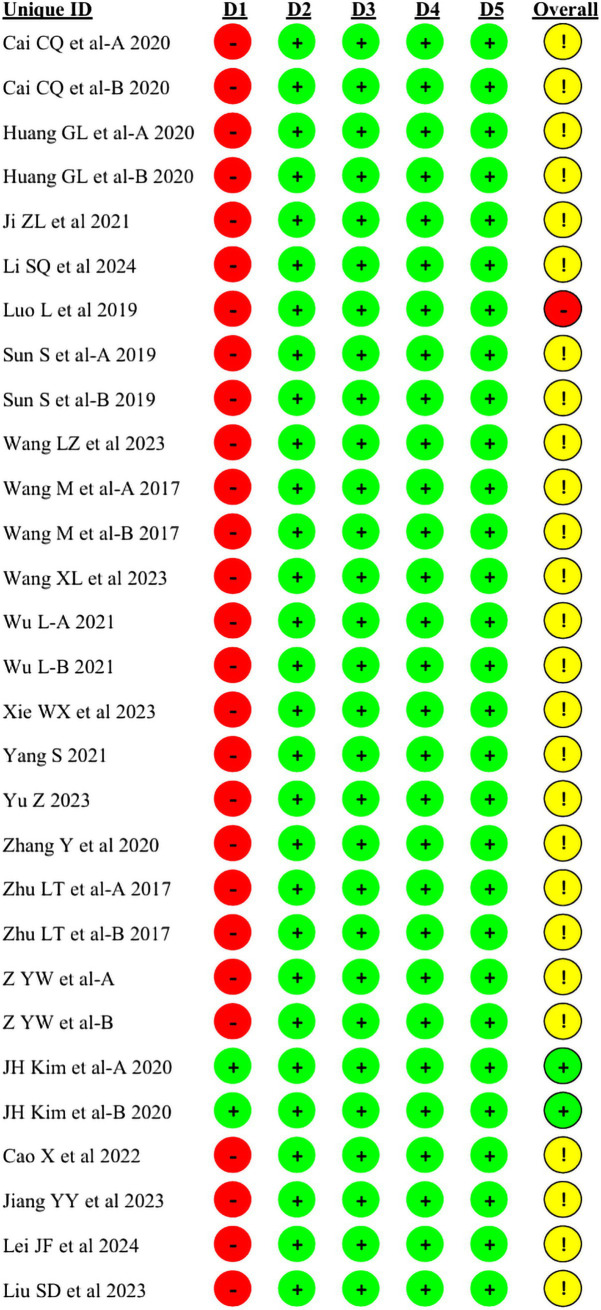
Risk of bias summary.

**Figure 3:**
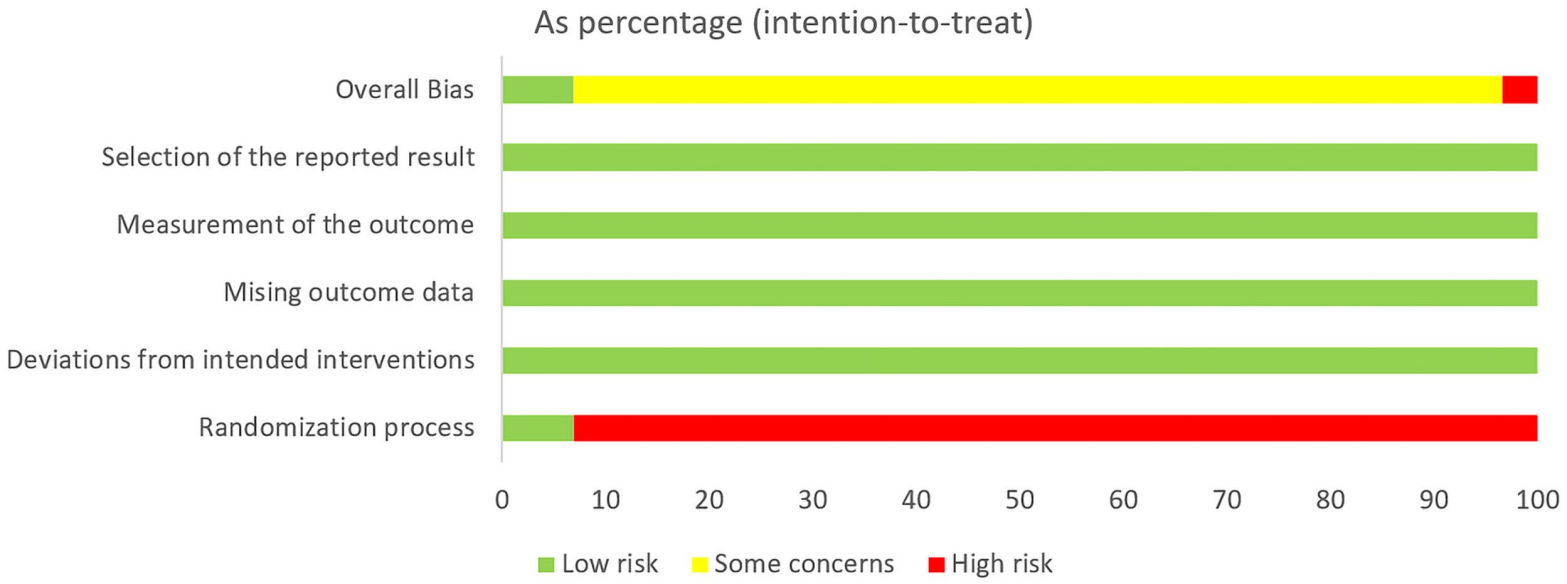
Risk of bias graph.

2 out of 29 studies were low risk, 1 study was high risk and the remaining 26 were medium risk. Due to the specificity of acupuncture therapy, the researchers were unable to achieve allocation concealment during the conduct of the trial, which was the main reason for reducing the quality of the studies. Despite the inability to achieve allocation concealment, most of the indicators in the studies were objective data and patients’ physical manifestations, so the results of the studies were reliable. Random number table method and computerized randomization were used for grouping, but 9 studies only referred to random assignment. 4 studies had patient dislodgement during the conduct of the study, which resulted in incomplete data, and the rest of the studies did not have patient dislodgement. The details are shown in Figures 2 and 3.

### 3.4 Main outcomes

#### 3.4.1 FMA-UE

**Figure 4:**
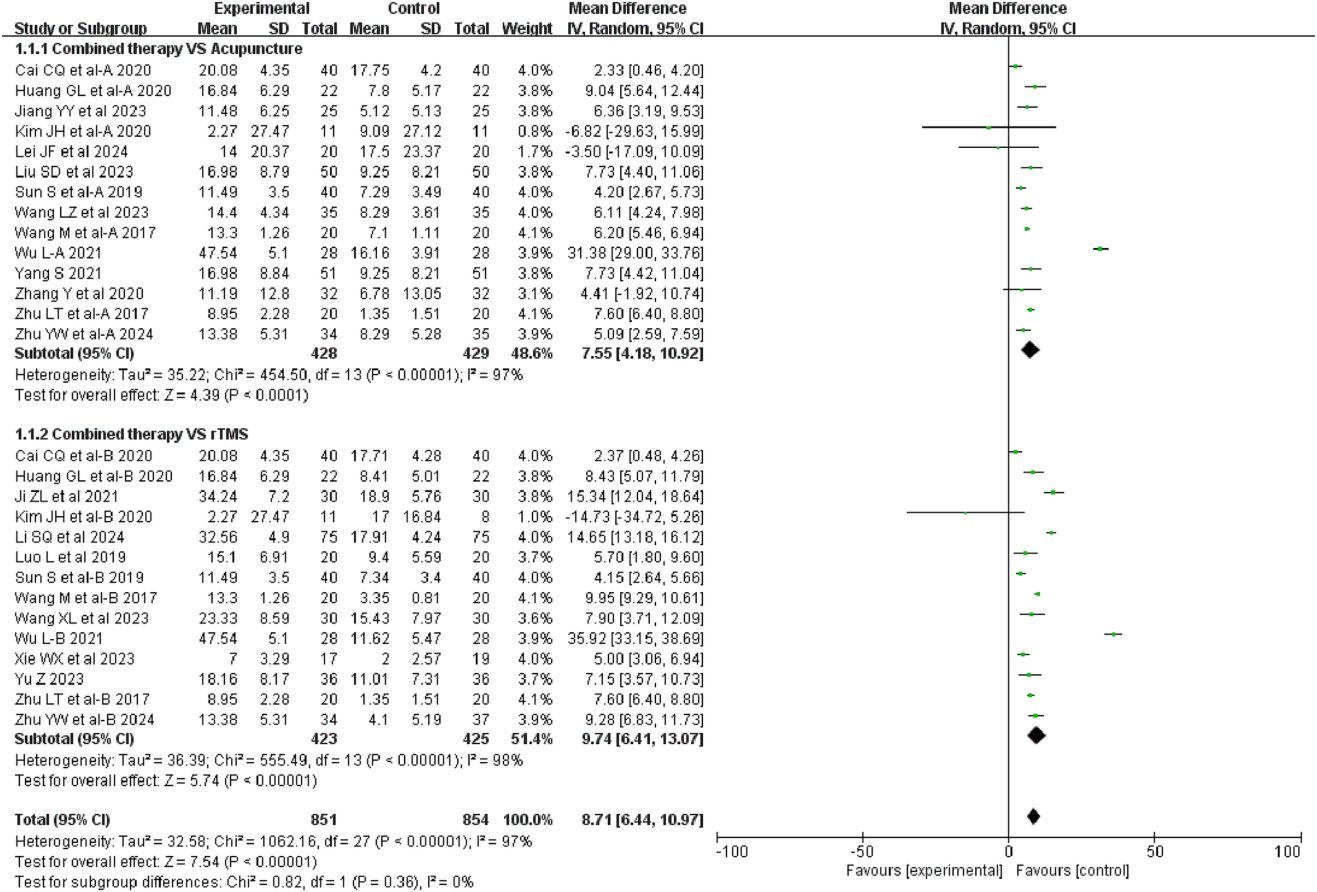
Forest plot of FMA-UE.

28 studies evaluated FMA-UE before and after treatment, involving a total of 1705 patients. Combination therapy was more efficacious in improving FMA-UE compared to acupuncture treatment (MD = 7.55, 95%CI: 4.18 ~ 10.92, I^2^ = 97%, *p* < 0.00001). Compared with rTMS treatment, the efficacy of combination therapy was more significant in improving FMA-UE (MD = 9.74, 95%CI: 6.41 ~ 13.07, I^2^ = 98%, *p* < 0.00001). The details are shown in Figure 4.

#### 3.4.2 MBI

**Figure 5:**
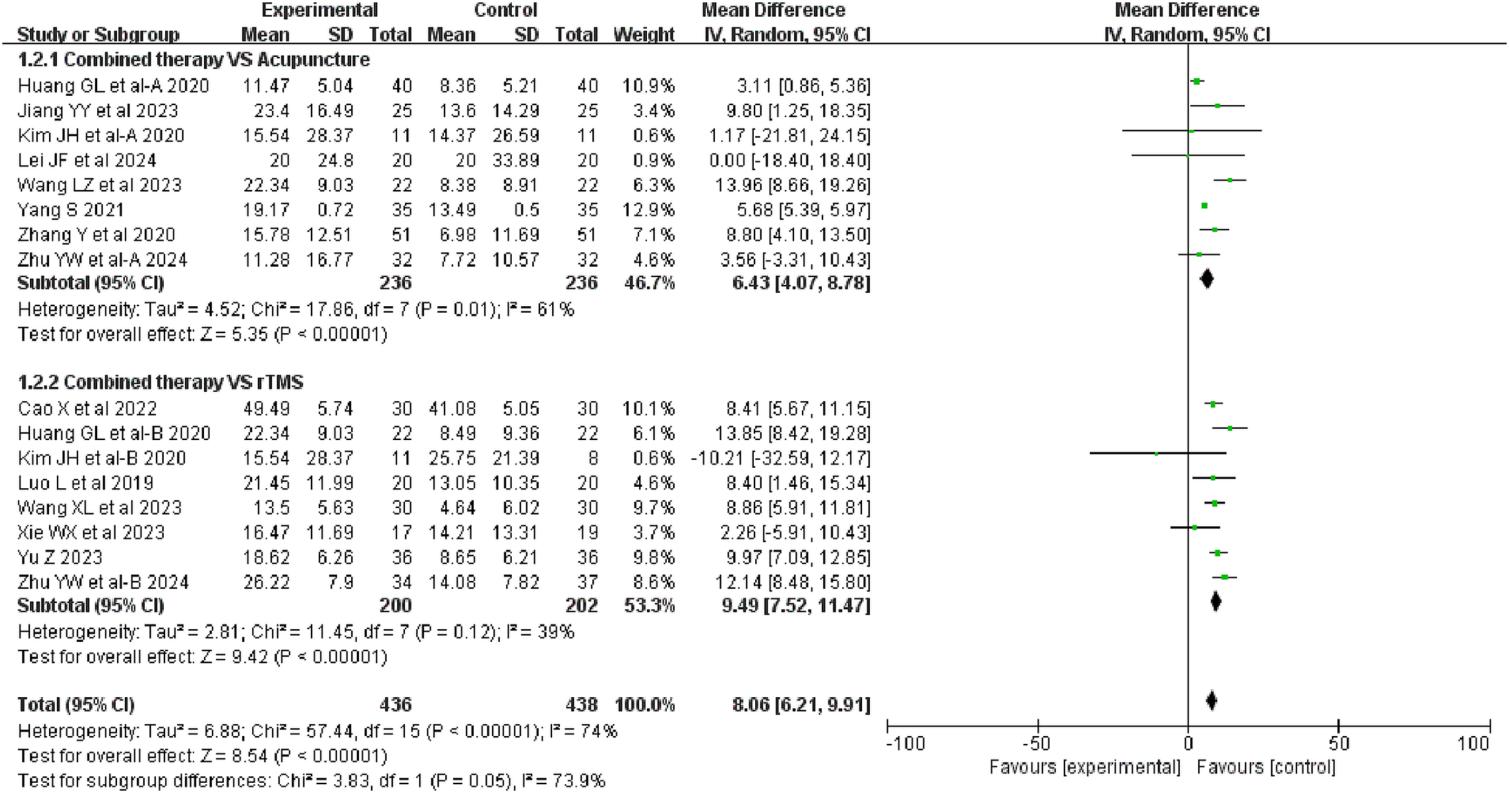
Forest plot of MBI.

16 studies evaluated MBI before and after treatment, involving a total of 874 patients. The efficacy of combination therapy was more pronounced in improving MBI compared to acupuncture treatment (MD = 6.43, 95%CI: 4.07 ~ 8.78, I^2^ = 61%, *p* = 0.01). Compared with rTMS treatment, the efficacy of combination therapy was more significant in improving MBI (MD = 9.49, 95%CI: 7.52 ~ 11.47, I^2^ = 39%, *p* = 0.12). The details are shown in Figure 5.

### 3.5 Secondary outcome indexes

#### 3.5.1 MAS

**Figure 6:**
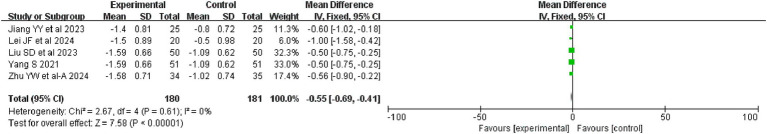
Forest plot of MAS.

5 studies comparing combination therapy with acupuncture in improving MAS included a total of 361 patients. The analysis found that combination therapy was more effective in improving MAS compared with acupuncture (MD = −0.55, 95% CI: −0.69 to −0.41, I^2^ = 0%, *p* = 0.61). For details, see Figure 6.

#### 3.5.2 NIHSS

**Figure 7:**
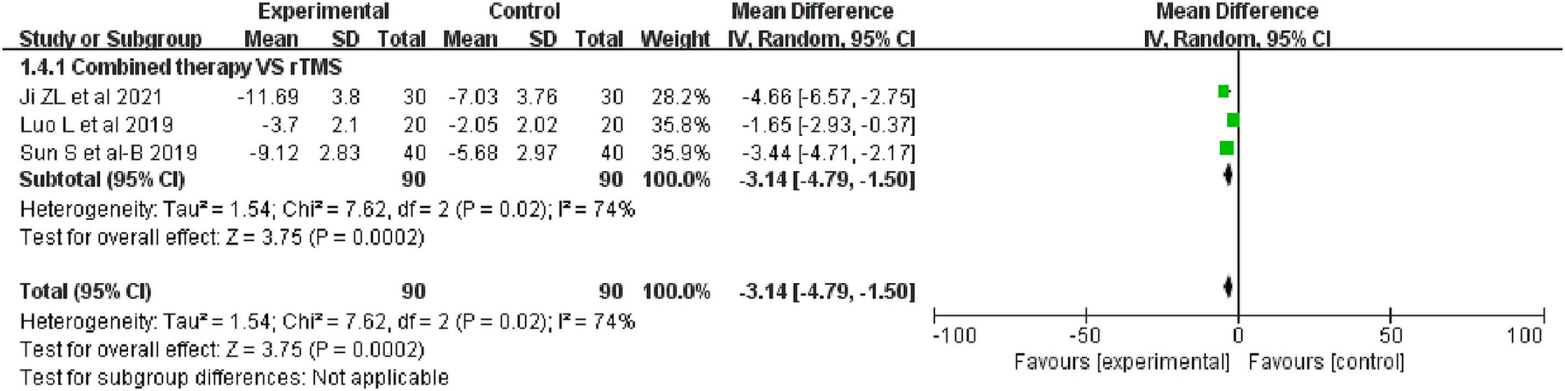
Forest plot of NIHSS.

3 studies comparing the difference in improvement in NIHSS between combination therapy and rTMS included a total of 180 patients. The analysis found that combination therapy was superior to rTMS therapy in improving NIHSS scores (MD = −3. 14, 95%CI: −4.79 to −1.5, I^2^ = 74%, *p* = 0.02), as detailed in Figure 7.

### 3.6 Publication bias

**Figure 8:**
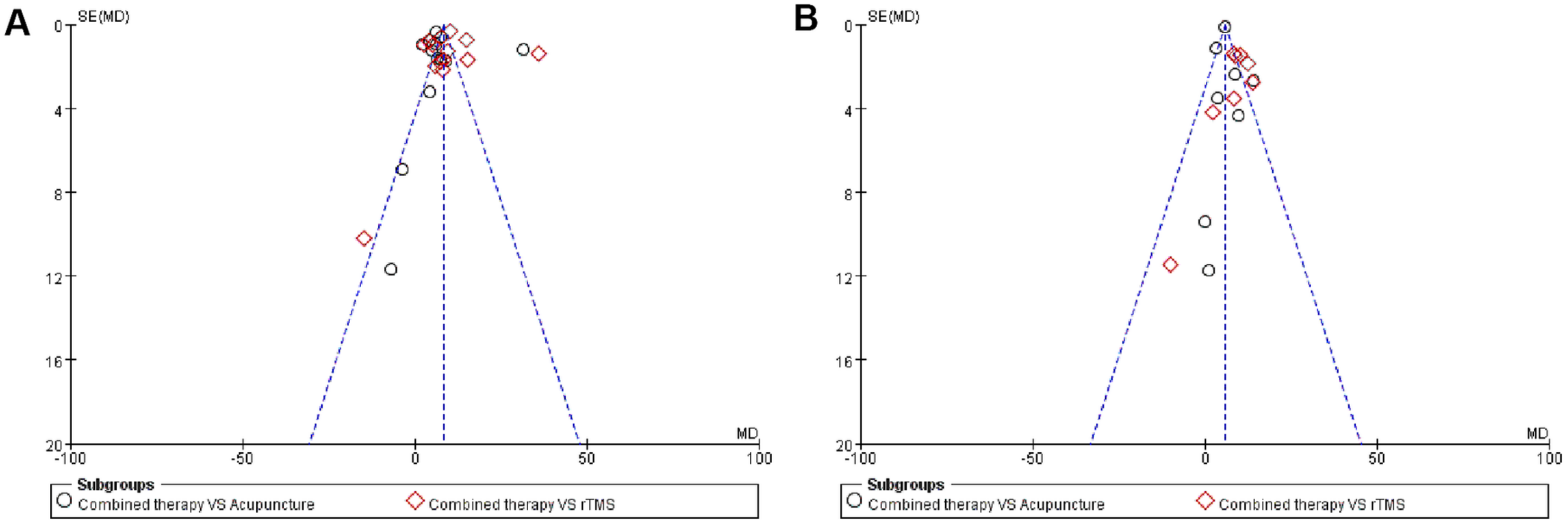
Publication bias. **(A)** Funnel plot of FMA-UE. **(B)** Funnel plot of MBI.

We applied funnel plots to assess publication bias for the two main outcome indicators, and the two funnel plots were slightly asymmetric, with some studies deviating from the funnel plots, indicating publication bias. A review of related studies found that the sample size included in some studies was too small, which may be the cause of the bias. The details are shown in Figure 8.

### 3.7 Quality assessment

We evaluated the available evidence using the GRADE tool, and the quality of the evidence for acupuncture combined with repetitive transcranial magnetic stimulation for the treatment of upper limb motor dysfunction after stroke was rated as “low.” Detailed information is shown in the Supplementary material.

## 4 Discussion

Stroke, as the second leading cause of death and the leading cause of disability worldwide, adversely affects patients and families, and puts a great deal of pressure on global health systems. Upper extremity motor dysfunction is one of the common complications of stroke, and stroke survivors who experience upper extremity motor function are often difficult to recover. How to promote the recovery of upper limb motor function and thus improve the mobility and quality of life of stroke survivors is a major issue at present. Addressing this issue may also alleviate the family burden as well as the social burden of stroke patients. Although previous studies have found acupuncture and rTMS to be positive in improving upper limb motor dysfunction in stroke patients, whether the synergistic application of the two has a synergistic effect has not been systematically organized. In this study, we analyzed 29 studies by collating 21 papers to investigate the effect of acupuncture combined with rTMS on improving upper limb motor function and quality of life after stroke, and observed the improvement of various indexes, which provided a reference for the clinical application of acupuncture combined with rTMS in the treatment of upper limb motor dysfunction after stroke.

28 studies assessed changes in the FMA-UE scale before and after treatment, and 16 studies assessed changes in the MBI scale before and after treatment. Combination therapies were more advantageous in improving FMA-UE compared with both acupuncture alone and rTMS intervention alone. The analyzed results for MBI were consistent with FMA-UE. We found significant heterogeneity in the results of the FMA-UE analyses. The same significant heterogeneity was found in the MBI analyses comparing the combination therapy with acupuncture alone. Our in-depth reading and comparison of the involved studies revealed that the involved studies differed in the selection of acupoints, method of acupuncture, duration of each intervention, and the selection of rTMS devices, which may account for the heterogeneity. In addition, Kim JH and Lei JF’s (20, 22) studies had contrary results to other studies, and in this study, the researchers did not find that the combination therapy was superior to acupuncture alone or rTMS intervention alone. Overall, the small sample size included in JH Kim and Lei JF’s studies and the presence of shedding during the treatment period make the results of this study potentially biased. We believe this study is responsible for the high heterogeneity.

The results of the Meta-analysis showed that the combination therapy was more advantageous in improving MAS and NIHSS compared with acupuncture alone or rTMS applied alone. In conclusion, the results of Meta-analysis showed that combination therapy was superior to acupuncture alone or rTMS alone intervention in improving upper limb motor dysfunction as well as neurological recovery and ability to live in stroke patients.

Currently, the effectiveness of acupuncture in improving post-stroke motor dysfunction is well recognized (25, 26). Researchers have explored the mechanisms by which acupuncture works and have proposed a number of different hypotheses (27–29). The clinical studies have found that acupuncture enhances stroke function and is effective in improving motor dysfunction after stroke. Clinical studies have found that acupuncture enhances bidirectional effective connectivity between the cerebellum and primary sensorimotor cortex in stroke patients, which may compensate for the weakened effective connectivity between cortical and subcortical areas during passive motor tasks, thus contributing to the improvement of motor performance in subacute stroke patients (30). Another study based on graph-theoretic analysis found that acupuncture could modulate the disruption patterns of subcortical ischemic poststroke whole-brain networks, revealing a potential mechanism for functional reorganization of poststroke brain networks involving acupuncture intervention (31). Animal studies have found that electroacupuncture enhances neural activity in brain regions associated with motor function in ischemic stroke rats, including the motor cortex, dorsal thalamus, and striatum (32). Another study similarly demonstrated that electroacupuncture can enhance neural activity in areas of the brain associated with motor function in ischemic stroke rats, including the motor cortex, dorsal thalamus, and striatum. Another study also demonstrated that electroacupuncture could enhance functional connectivity between the left motor cortex and motor-related brain regions (including motor cortex, sensory cortex, and striatum) in ischemic stroke rats, which in turn improved motor deficits and enhanced brain function recovery (33). Additional studies have shown that the combination of scalp acupuncture and LF-rTMS is more advantageous than scalp acupuncture alone in promoting cerebral white matter repair in stroke patients, which may be one of the ways to promote the recovery of upper extremity function in stroke patients (34).

rTMS is also widely used in the treatment of stroke and is thought to enhance brain plasticity in the stroke organism, with positive implications for the rehabilitation of stroke survivors (30). The results of a meta-analysis showed that rTMS can improve balance and activities of daily living in stroke patients, and is an effective and safe technology (31). A study A study found that rTMS inhibited pro-inflammatory M1 activation (Iba1/CD86) and ameliorated anti-inflammatory M2 activation (Iba1/CD206) in the peri-infarct zone of ischemic stroke mice, and improved motor dysfunction and neuroinflammation after brain injury in mice by modulating microglial cell polarization (32). A recent meta-analysis revealed the effectiveness of rTMS in improving upper limb motor deficits after stroke and suggested the need for comprehensive consideration of multiple factors in analyzing the effectiveness of rTMS (33).

Based on the favorable results achieved by acupuncture and rTMS alone, clinicians are gradually combining acupuncture and rTMS in the treatment of stroke survivors. The results of this study also confirm that the combination of acupuncture and rTMS is better than acupuncture alone or rTMS alone in improving upper limb motor dysfunction after stroke, but again there are some key factors that need to be further considered, such as the parameter settings of rTMS, the selection of points and modalities of acupuncture, and the stage of the patient’s disease. The literature included in this study used LF-rTMS for intervention, and thus failed to analyze the differences in clinical efficacy of rTMS at different frequencies. In addition, the current protocols for the treatment of upper limb motor dysfunction after stroke by acupuncture are based on the experience of clinicians, and there is no uniform standardized protocol. The duration of the patient’s illness and the type of stroke diagnosis are also important factors affecting clinical efficacy, and is the treatment program consistent at different stages? Is there consistency in the selection of acupuncture points and the degree of stimulation of rTMS? These are all issues that need to be resolved urgently. Therefore, more clinical studies are needed to further explore the optimal therapeutic strategy for the combined application of acupuncture and rTMS.

## 5 Conclusion

The results of this study suggest that acupuncture combined with rTMS can be more effective in improving patients’ upper limb motor function and quality of life, and help to promote the recovery of neurological function, compared with acupuncture alone or rTMS applied alone. However, due to the high degree of heterogeneity in the analysis of some of the included studies, which might have had an impact on the results, the results of the current analysis still need to be treated with caution, and future studies should further strengthen the experimental design and reduce the heterogeneity by conducting studies with large samples in order to further explore the reliability of this result. However, due to the limitations of this study, this result needs further validation.

## Data Availability

The original contributions presented in the study are included in the article/Supplementary material, further inquiries can be directed to the corresponding author.

## Footnotes

^1^ http://gdt.gradepro.org/app#projects
